# Effect of Altering Dietary *n*-6:*n*-3 Polyunsaturated Fatty Acid Ratio with Plant and Marine-Based Supplement on Biomarkers of Bone Turnover in Healthy Adults

**DOI:** 10.3390/nu9101162

**Published:** 2017-10-24

**Authors:** Sujatha Rajaram, Ellen Lan Yip, Rajneesh Reghunathan, Subburaman Mohan, Joan Sabaté

**Affiliations:** 1 Center for Nutrition, Healthy Lifestyle and Disease Prevention, School of Public Health, Loma Linda University, Loma Linda, CA 92350, USA; ellenlannguyen@gmail.com (E.L.Y.); jsabate@llu.edu (J.S.); 2 Musculoskeletal Disease Center, Loma Linda VA Healthcare Systems, Loma Linda, CA 92357, USA; reghu.rajneesh@gmail.com (R.R.); subburaman.mohan@va.gov (S.M.); 3 Department of Medicine, Loma Linda University, Loma Linda, CA 92350, USA

**Keywords:** dietary *n*-3 fatty acids, bone turnover, peroxisomal proliferator activated receptor γ, ALA, EPA/DHA

## Abstract

Although there is accumulating evidence for a protective role of *n*-3 polyunsaturated fatty acids (*n*-3 PUFAs) on bone health, there are limited studies that examine the effect of altering dietary *n*-6:*n*-3 PUFA ratio with plant and marine sources of *n*-3 PUFA on bone health. Healthy adults (*n* = 24) were randomized into an eight-week crossover study with a four-week washout between treatments, with each subject consuming three of four diets. The four diets differed in the dietary *n*-6:*n*-3 PUFA ratios and either had an algal oil supplement added or not: (Control diet (10:1); α-linolenic acid (ALA) diet (2:1); Eicosapentaenoic acid/Docosahexaenoic acid (EPA/DHA) diet (10:1 plus supplement (S) containing EPA/DHA; Combination diet (2:1 + S)). The supplement was microalgae oil that provided 1 g EPA + DHA/day. Flaxseed oil and walnuts provided 8.6 g of ALA/day in the 2:1 diets. Serum levels of c-telopeptide (CTX), procollagen Type I *N*-terminal peptide, and osteocalcin showed significant correlation with age but none of the bone markers or peroxisomal proliferator-activated receptor-γ mRNA expression was significantly different between the diets. Serum CTX was negatively associated with red blood cell membrane linoleic acid and ALA and positively associated with membrane DHA. Neither altering dietary *n*-6:*n*-3 PUFA ratio from a 10:1 to a 2:1 ratio nor adding EPA/DHA supplement significantly changed bone turnover in the short term in healthy adults.

## 1. Introduction

*N*-3 polyunsaturated fatty acids (*n*-3 PUFAs) confer many health benefits including the prevention of cardiovascular, cardiometabolic, and other chronic diseases as well as the reduction of inflammation [1,2,3]. A limited number of human studies suggest that *n*-3 PUFAs play an important role in bone metabolism and may represent a potentially useful non-pharmacological therapeutic strategy to prevent bone loss and reduce the risk of osteoporosis [4,5]. The essential *n*-3 PUFA, α-linolenic acid (ALA) cannot be synthesized by humans, while eicosapentaenoic acid (EPA) and docosahexaenoic acid (DHA) can be generated from ALA, although the conversion rate is low. Western diets are low in *n*-3 PUFA and high in *n*-6 PUFA, which makes for a high dietary *n*-6:*n*-3 PUFA ratio. The health attributes of *n*-3 PUFA is due to the direct effects of ALA, or the conversion of ALA to EPA and DHA and/or the decrease in the *n*-6:*n*-3 PUFA ratio. Animal studies have demonstrated the protective role of fish oil in preventing bone loss in mice following ovariectomy [6,7], with a marked increase in mineral apposition rate. In many of these studies, *n*-3 fatty acids or a lower ratio of *n*-6:*n*-3 PUFA show a positive influence on bone. Accordingly, populations known to consume high amounts of *n*-3 PUFA-rich fish, such as the Japanese and Greenland Eskimos, have lower rates of osteoporosis [8]. *N*-3 PUFAs are thought to mediate their actions by regulating the fatty acid composition of skeletal cells [9,10]. Since the concentration of ALA in mammalian cell membranes is extremely low and makes up less than 0.5% of total fatty acids in plasma phospholipids, the specific functional and protective effects of ALA are attributed to its conversion to longer chain *n*-3 fatty acids, EPA and DHA [11,12,13].

Intervention trials with PUFAs in skeletal metabolism in humans are limited, and findings are controversial [14,15,16,17]. One potential explanation for this is that the skeletal effects of *n*-3 fatty acids may depend on the type, dose, and duration of treatment. Typical Western diets are associated with higher *n*-6:*n*-3 PUFA ratios, whereas, it is the low *n*-6:*n*-3 ratios that are correlated with optimal health and decreased risk of disease [4,5]. Griel et al. [4] showed that plant sources of *n*-3 PUFA lower bone resorption, especially when the background *n*-6:*n*-3 ratio is low (1.6:1). Studies investigating the role of marine *n*-3 PUFA (EPA/DHA) in the context of low and high *n*-6:*n*-3 PUFA ratios and comparing plant versus marine *n*-3 PUFAs in preventing bone loss in humans are sparse. Thus, the objective of this study was to examine the effect of altering the dietary *n*-6:*n*-3 PUFA ratio from 10:1 to 2:1 with and without adding a supplement of EPA/DHA.

## 2. Materials and Methods

### 2.1. Study Design

This study was part of a larger intervention trial assessing the changes in red blood cell (RBC) membrane fatty acid composition when the dietary *n*-6:*n*-3 fatty acid is altered from a ratio of 10:1 to 2:1 by adding plant- and marine-based supplements [18]. This aim of this study was to assess the effects of altering the dietary *n*-6:*n*-3 fatty acid ratio on biochemical markers of bone turnover and gene expression in healthy adults. This study was a single-blind, randomized, 4 × 3 incomplete crossover trial including four diets: (1) Control diet (*n*-6:*n*-3 ratio of 10:1, low in ALA, EPA/DHA); (2) EPA/DHA diet (10:1 plus supplement (S) of algal oil, low in ALA, high in EPA/DHA); (3) ALA diet (2:1, high ALA from walnuts and flaxseed oil, low EPA/DHA); and (4) Combination diet (2:1 + S, high in ALA and EPA/DHA). There was an initial one-week run-in phase to assess each participant’s adherence to the dietary protocol. Study periods were eight weeks each and included a washout of four–six weeks between treatments. All meals were provided to the subjects and dietary compliance was assessed by the examination of individual participant diaries and through direct observation by research staff at each meal on campus. The complete study design and subject protocol have been previously published [18].

### 2.2. Subjects

The total number of participants completing all three diet periods was 24 (15 females and 9 males, age 42 ± 3 years). Participants were recruited from the Loma Linda area, including nearby hospitals and colleges. Participants that met all study criteria and received the highest commitment scores were selected. All selected subjects signed the informed consent approved by the Institutional Review Board at Loma Linda University (Loma Linda, CA, USA).

Subjects were included in the study if they had: (a) no prior affliction with hypertension, atherosclerosis, or other metabolic diseases; (b) serum cholesterol levels between 4.2–7.8 mmol/L; (c) serum triglyceride below 3.4 mmol/L; (d) body mass index below 30 kg/m^2^; (e) stable weight within the past six months; (f) no intake of serum lipid-altering medications; (g) age range between 20 and 70 years; (h) no known food allergies to walnuts, flaxseed oil, or microalgae oil. All participants were non-smokers and maintained the same level of physical activity throughout the study as was established at their baseline.

### 2.3. Study Diets

There were four diets in all. Two diets had an *n*-6:*n*-3 fatty acid ratio of 10:1 (Control diet without supplement and EPA/DHA diet with supplementation, 1.40/5.04 g EPA/DHA from microalgae oil/week). There were two other diets with an *n*-6:*n*-3 fatty acid ratio of 2:1 (ALA diet, 42–49 g flaxseed oil/week + 10 g walnuts, 3 times/week and Combination diet with both ALA (42–49 g flaxseed oil/week + 10 g walnuts, 3 times/week) and supplementation, 1.40/5.04 g EPA/DHA/week). This was a rigorously controlled feeding study with all meals provided to participants by the research staff. Participants consumed dinners on the premises at Loma Linda University Campus, with all breakfast, lunch, and snacks provided as take out. All Saturday meals were packed and distributed to participants during Friday dinner. In order to increase dietary compliance, all dine-in meals were monitored by at least one senior investigator.

Menus were designed for seven levels of energy intake, ranging from 1500 to 3000 kcal/day to accommodate the eucaloric requirements of the subjects and has been described previously [18]. The main sources of *n*-3 fatty acid-rich foods were EPH/DHA-rich microalgae oil and ALA-rich flaxseed oil and walnuts. All menu plans adhered to a nine-day weekday and two-day weekend menu cycle with lacto-ovo vegetarian meals provided throughout the study.

### 2.4. Data Collection and Analyses

Each participant had fasting blood drawn at baseline and at the end of each diet period. Blood samples were collected at the standardized time of the day, i.e., 6:30 a.m. to 8:30 a.m. The variation between subjects was expected to be much higher than the minimal variation caused by sample collection in a 24-h time window [19]. Blood draw clinics were conducted at the Nutrition Assessment Laboratory and serum samples were stored at −80 °C. Lipomics Laboratory (Sacramento, CA, USA) measured RBC membrane fatty acid composition at the end of each treatment period.

Biochemical determination of all serum bone markers was carried out in duplicate runs after each experimental diet. Bone resorption marker c-telopeptide (CTX) was measured using the Serum Crosslaps ELISA Assay (Immunodiagnostic Systems Limited, Boldon, UK) that quantifies the degradation products of C-terminal telopeptides of Type 1 collagen in human serum. The intra- and inter-assay coefficients of variation were <6% and <10%, respectively. Bone formation marker procollagen type I N-terminal propeptide (P1NP) was measured using the UNiQ P1NP RIA (Orion Diagnostica, Espoo, Finland). This radioimmunoassay uses both labeled and unlabeled P1NP to competitively bind to limited sites located on polyclonal rabbit anti-P1NP antibody. The intra- and inter-assay coefficients of variation were 6.5–10.2% (12–173 µg/L) and 6.0–9.8% (12–167 µg/L), respectively. Bone formation marker osteocalcin (OC) was measured using N-MID Osteocalcin ELISA (Nordic Bioscience Diagnostics, Herlev, Denmark). This assay measures the N-Mid fragment region of OC in human serum. The intra- and inter-assay coefficients of variation were <4% and <7%, respectively. Insulin-like growth factor 1 (IGF-1) was measured using the assay IGF-1 ELISA (Immunodiagnostic Systems Limited, Boldon, UK), which quantifies the amount of this polypeptide in human serum. This assay uses a highly specific purified polyclonal sheep antibody and a high affinity labeled monoclonal anti-IGF-1 with horseradish peroxidase. The intra- and inter-assay coefficients of variation were <4% and <7%, respectively.

At the end of each diet period, samples of subcutaneous tissue were collected from the abdominal region and used for measurement of peroxisomal proliferator activated receptor-gamma (PPAR-γ) mRNA levels by real-time polymerase chain reaction (PCR) with actin as an internal standard.

### 2.5. Statistical Analyses

A trained statistician using SAS software, version 9.1 (SAS Institute Inc., Cary, NC, USA) performed the statistical analyses. Data are reported as least squares mean + standard error. A mixed-effect model was used that included a random-effect term for subjects nested in sequence, a fixed-effect term for period and treatment, and a covariate term representing the amount of specific *n*-3 fatty acids (ALA and/or EPA/DHA) in each respective diet. The Kenward-Roger method and Tukey-Kramer HSD tests were performed to estimate denominator degrees of freedom for tests of fixed effects and to evaluate significant pair-wise differences among the diets, respectively. A mixed model approach was also used to evaluate the association between bone markers (CTX, PINP, and OC) with individual RBC membrane fatty acids (Linoleic (LA), ALA, EPA, and DHA), adjusting for treatments and period effect. A pre-determined level of statistical significance was set at *p* < 0.05. The expression changes of PPARγ mRNA levels in different subjects were standardized to expression levels of the housekeeping gene, actin, and comparisons for different study diets made using student’s *t*-test.

## 3. Results

### 3.1. Nutrient Analyses and Dietary Compliance

The nutrient composition of treatment diets from chemical analyses (Covance Laboratories, Madison, WI, USA) revealed that the percentage of total fat (≈30%), saturated fat (<10%), and trans fatty acids (<1%) were in the appropriate range [18]. The *n*-6:*n*-3 PUFA ratio for the ALA and combination diets was 2.5:1, and for the control and EPA/DHA diets was approximately 9.3:1. Both ratios were extremely close to the planned ratios of 2:1 and 10:1 for the ALA/combination diet and control/EPA+DHA diets, respectively [19]. Dietary compliance assessed through RBC fatty acid composition for each participant at the end of diet treatment indicated excellent adherence to dietary protocol as described previously [18].

### 3.2. N-3 Fatty Acids and Bone Markers

Mean serum CTX, PINP, and OC concentrations among the Control, EPA/DHA, ALA, and Combination diets are reported in Table 1. There was no significant diet effect or pair-wise differences among treatment diets, even after adjusting for age and gender (*p* > 0.05).

### 3.3. Correlation between Bone Markers, n-3 Fatty Acids, and Age

A mixed model approach was used to examine the association between bone markers (CTX, PINP, and OC) and individual *n*-3 fatty acids (Table 2). There was a significant negative association between serum CTX with RBC membrane LA (*p* = 0.0143) and ALA (*p* = 0.0477). There was a significant positive association with serum CTX and RBC membrane DHA (*p* = 0.0385). Even after adjusting for gender, results were significant.

A mixed model approach was also used to investigate an association between age and bone markers (CTX, PINP, and OC). There was a significant negative association between age and bone markers CTX (*p* < 0.0001), P1NP (*p* = 0.0006), and OC (*p* = 0.0019) (Table 2).

### 3.4. Correlation between Bone Markers and IGF-1

**Figure 1:**
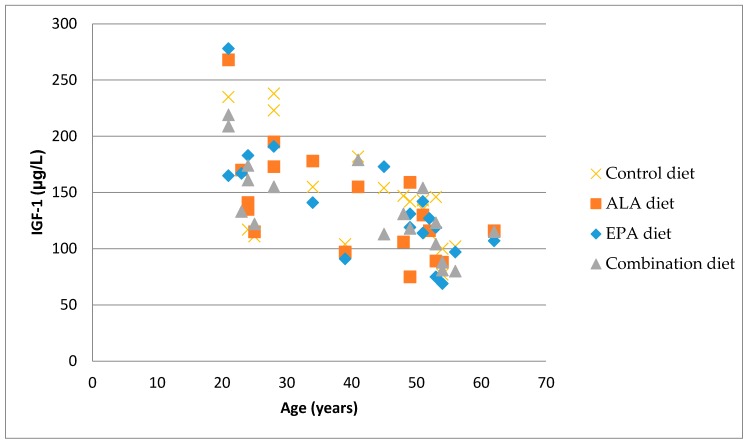
The association between serum IGF-1 levels and age in different diet groups.

After adjusting for age and gender, there were no significant associations between CTX, PINP, or OC and serum IGF-1 (*p* > 0.05). However, there was a significant negative association between serum IGF-1 and age (*p* < 0.0001) (Figure 1). The correlation coefficient between serum IGF-1 levels and age for the Control (*r*^2^ = 0.3459), EPA/DHA (*r*^2^ = 0.5922), ALA (*r*^2^ = 0.4174), and Combination (*r*^2^ = 0.5382) treatment diets in healthy adults are significant at *p* < 0.001 (*n* = 24). 

### 3.5. Gene Expression

There were no significant changes in PPAR γ expression between the four different diets (Table 3). 

## 4. Discussion

We observed that altering the *n*-6:*n*-3 fatty acid ratio from 10:1 to 2:1 by increasing ALA (8.6 g of ALA) or by adding a supplement (1 g EPA + DHA/day) to either the 10:1 or 2:1 diet in the short term (eight weeks) did not alter serum bone markers or PPAR-γ gene expression in healthy adults. While these amounts promote cardioprotective effects [1,20], it appears that this dose may not be sufficient to influence bone turnover in healthy subjects. Previously, a six-week feeding trial by Griel et al. [4] providing a significantly higher dose (17 g ALA from walnuts and flaxseed oil) showed a significant reduction in bone resorption marker *N*-telopeptide. Lack of results from our cohort may be partly due to a lower ALA intake even in our 2:1 diet groups. Alternatively, the duration of intervention may have been too short to see changes in bone turnover markers in a relatively young, healthy adult cohort. 

A low dietary ratio of *n*-6:*n*-3 PUFA has been correlated with increased bone mineral density in the hip in older adults and in the spine for healthy young men [5,21,22]. Weiler et al. [23] showed that diets with low *n*-6:*n*-3 ratios resulted in higher plasma DHA levels and decreased bone resorption in growing piglets. In our study, just altering the ratio from 10:1 to 2:1 did not increase RBC membrane DHA levels [18]. Although the 2:1 ALA diet had a high amount of ALA, conversion of ALA to DHA was poor. When the algal supplement containing EPA/DHA was added to the 2:1 diet (combination diet), then the DHA in RBC membrane increased significantly. It was still not sufficient to alter bone turnover markers. It appears that in healthy adults, the bone turnover rate is low and in order for diet-induced changes to occur, either a longer duration of intervention or a higher dose of intervention may be necessary. 

The bone-protective effects observed with a low dietary *n*-6:*n*-3 ratio [4,5,21] may be attributed to a lower concentration of eicosanoids arising from the *n*-6 fatty acids pathway [24]. Evidence suggests that eicosanoids derived from arachidonic acid, a byproduct of *n*-6 fatty acid metabolism, have been linked to numerous inflammatory and autoimmune disorders [11]. We observed a significant negative association between serum CTX and RBC membrane LA and ALA. Other research also supports that lower dietary ratios of *n*-6:*n*-3 fatty acids protect bone [9]. The plausible mechanism by which tissue levels of ALA could influence bone resorption is via prostaglandin E2 (PGE2), a primary eicosanoid that affects bone metabolism and inhibits the activation of receptor-activated nuclear-kappa B ligand (RANKL), an important growth factor that promotes osteoclastogenesis [25]. In a nine-week animal study by Mollard et al. [26], ALA-rich flaxseed oil diet significantly reduced PGE2 levels. Dietary intake of ALA may exert an anabolic effect on bone through lowering concentrations of PGE2 [27]. Although the RBC ALA levels increased both by increasing ALA (2:1 diet) and by adding a supplement (10:1 + S and 2:1 + S diets) in our study, it is likely that it was insufficient to modify PGE2 or other inflammatory markers. Perhaps individuals with elevated inflammation at baseline may better respond to *n*-3 PUFA with respect to markers of bone formation and resorption. This needs to be explored in future studies. 

Higher endogenous DHA helps reduce bone resorption. Serum phospholipid DHA levels were positively associated with total bone mineral density in healthy men between 16 and 22 years of age [21]. In animal studies, a high PUFA diet incorporating DHA-rich single cell oil supplementation increased femur bone mineral density and diets using lower ratios of *n*-6:*n*-3 fatty acids observed less bone resorption [27,28]. These findings suggest that high DHA supplementation may be beneficial and may help promote bone conservation. In contrast, we found a positive correlation between DHA levels and bone resorption, which could be due to the different age group of subjects used in this study. There was a significant negative association between age and bone markers CTX, P1NP, and OC. These findings are in agreement with other studies showing a biphasic effect of age on bone remodeling in specific age groups [29,30]. Bone resorption and formation markers decline with age, reaching their lowest levels between 30 and 50 years.

The majority of participants in this study were within this age range, with only 2 participants over 55 years of age (mean age = 42). After the age of 50, markers of both bone formation and resorption increase with resorption, exceeding the formation that is caused by lowering sex hormone levels [31]. There was also a significant negative association between age and IGF-1, which is in agreement with what others have observed [32]. Veldhuis et al. [33] showed that IGF-1 concentrations can actually decrease by more than 50% in healthy older adults. The effects of the different dietary PUFA ratios on serum IGF binding protein levels may be worth exploring.

Previously, it has been shown that *n*-3 fatty acids modulate the expression of PPARγ [34]. However, we did not find a significant difference in the subcutaneous tissue PPARγ among the four different diets. This lack of difference could be due to the use of subcutaneous rather than adipose tissue. While dietary fatty acids including *n*-3 PUFAs may have beneficial effects on bone, regulators of postprandial skeletal fatty acid flux need to be identified. One of the proposed regulators is lipoprotein lipase (LPL), involved in the metabolism of triglyceride-rich lipoproteins [35]. Since *n*-3 PUFAs are known to lower triglyceride levels through improved clearance of these lipids by activating LPL, future studies should consider exploring this relationship.

## 5. Conclusions

Epidemiological and intervention studies have shown that increasing *n*-3 PUFAs (ALA, EPA/DHA) and/or lowering the dietary *n*-6:*n*-3 PUFA ratio may have bone protective effects [4,17,21,22]. However, our study on healthy adults did not show any change in bone formation or resorption markers, even when the dietary *n*-6:*n*-3 PUFA ratio was altered from 10:1 to 2:1 using ALA-rich food sources or when a supplement containing EPA/DHA was added to the two diets. A low rate of bone turnover in these relatively young and healthy adults may be one of the reasons for the lack of favorable results. There is still merit to exploring the role of *n*-3 fatty acids on bone metabolism since these fatty acids clearly play a role in bone remodeling and bone microstructure. However, due consideration must be given to the type, dose, and duration of intervention and to the target population. Comparing the different sources of *n*-3 PUFAs is relevant since it will inform our dietary choices. From our current study, it is evident that incorporating plant sources of *n*-3 PUFA (ALA) can help reduce the dietary *n*-6:*n*-3 PUFA ratio and increase RBC EPA levels significantly. Whether or not these translate to protective effects on the bone metabolism of healthy adults’ remains to be further explored.

## Figures and Tables

**Table 1:** Mean concentrations of serum bone markers at the end of each experimental diet ^1^.

Bone Markers			
Diet	CTX (ng/mL)	PINP (µg/L)	OC (ng/mL)
Control (10:1) ^2^	0.538 (0.041)	54.68 (2.96)	18.46 (1.13)
EPA/DHA (10:1 + S)	0.480 (0.041)	51.44 (2.96)	18.01 (1.13)
ALA (2:1)	0.588 (0.041)	50.10 (2.96)	16.34 (1.13)
Combination (2:1 + S)	0.583 (0.041)	50.89 (2.96)	16.91 (1.13)

^1^ Least Square Mean (Standard Error). There was no significant diet effect among the experimental diets (*p* > 0.05). ^2^ *n*-6:*n*-3 ratio. CTX-C-telopeptide, PINP-procollagen type I *N*-terminal propeptide, OC-Osteocalcin, S-supplement containing microalgae oil that provided EPA/DHA of 1 g/day.

**Table 2:** Association between bone markers with individual *n*-3 fatty acids and age ^1^.

Bone Markers	*n*-3 Fatty Acid	Estimate	*p*-Value
CTX	LA	−0.058 (0.023)	0.0143
	ALA	−0.419 (0.208)	0.0477
	EPA	0.068 (0.133)	NS
	DHA	0.038 (0.018)	0.0385
	Age	−0.017 (0.00345)	<0.0001
P1NP	LA	0.083 (1.50)	NS
	ALA	11.18 (13.13)	NS
	EPA	−11.46 (8.19)	NS
	DHA	−1.50 (1.13)	NS
	Age	−0.909 (0.225)	0.0006
OC	LA	−0.903 (0.614)	NS
	ALA	−2.98 (5.32)	NS
	EPA	−1.81 (3.36)	NS
	DHA	−0.102 (0.463)	NS
	Age	−0.429 (0.122)	0.0019

^1^ Even after adjusting for age and gender, results were still significant. NS-Not Significant.

**Table 3:** Expression of PPARγ mRNA in the subcutaneous tissue.

Diets	Fold Change ± SEM	*p*-Value
10:1 versus 2:1	1.72 ± 0.26	0.19
10:1 versus 10:1 + S	2.02 ± 0.32	0.11
10:1 versus 2:1 + S	2.20 ± 0.55	0.38
2:1 versus 10:1 + S	1.40 ± 0.22	0.55
2:1 versus 2:1 + S	1.52 ± 0.38	0.85
10:1 + S versus 2:1 + S	1.32 ± 0.33	0.55

Values are fold change versus actin mRNA levels. SEM-Standard error of the mean.
